# A Computational Investigation of In Vivo Cytosolic Protein Delivery for Cancer Therapy

**DOI:** 10.3390/pharmaceutics13040562

**Published:** 2021-04-15

**Authors:** Camilo Torres, Simon Dumas, Valentina Palacio-Castañeda, Stéphanie Descroix, Roland Brock, Wouter P. R. Verdurmen

**Affiliations:** 1 Department of Biochemistry, Radboud Institute for Molecular Life Sciences (RIMLS), Radboud University Medical Center, Geert Grooteplein 28, 6525 GA Nijmegen, The Netherlands; juancamilo.torresBonilla@radboudumc.nl (C.T.); valentina.palacio-castaneda@radboudumc.nl (V.P.-C.); roland.brock@radboudumc.nl (R.B.); 2 Physico-Chemistry Curie, Institut Curie, PSL Research University, CNRS UMR168, Sorbonne University, 75005 Paris, France; simon.dumas@curie.fr (S.D.); stephanie.descroix@curie.fr (S.D.)

**Keywords:** binding-site barrier, drug delivery modelling, cytosolic protein delivery, peptide delivery, targeted protein degradation, cancer therapy

## Abstract

The ability to specifically block or degrade cytosolic targets using therapeutic proteins would bring tremendous therapeutic opportunities in cancer therapy. Over the last few years, significant progress has been made with respect to tissue targeting, cytosolic delivery, and catalytic inactivation of targets, placing this aim within reach. Here, we developed a mathematical model specifically built for the evaluation of approaches towards cytosolic protein delivery, involving all steps from systemic administration to translocation into the cytosol and target engagement. Focusing on solid cancer tissues, we utilized the model to investigate the effects of microvascular permeability, receptor affinity, the cellular density of targeted receptors, as well as the mode of activity (blocking/degradation) on therapeutic potential. Our analyses provide guidance for the rational optimization of protein design for enhanced activity and highlight the importance of tuning the receptor affinity as a function of receptor density as well as the receptor internalization rate. Furthermore, we provide quantitative insights into how enzymatic cargoes can enhance the distribution, extent, and duration of therapeutic activity, already at very low catalytic rates. Our results illustrate that with current protein engineering approaches, the goal of delivery of cytosolic delivery of proteins for therapeutic effects is well within reach.

## 1. Introduction

In comparison to the rapid growth of the arsenal of protein-based therapies targeting extracellular receptors, the development of therapies in which proteins address an intracellular target lags far behind. Only three have been approved so far: the recombinant immunotoxins denileukin diftitox (Ontak), tagraxofusp, and moxetumomab pasudotox [1,2,3]. All act by delivering catalytic protein domains that block protein synthesis. These isolated examples made it into the clinic because they combine an extremely high potency with relatively well-accessible targets in T cell lymphomas and B cell leukemias. This particular set of properties is shared with many more similar agents currently in clinical development [4]. In contrast, few therapeutic proteins acting inside the cell are far in development for solid tumors or other diseases in which cells in a tissue need to be reached [4].

Beyond recombinant immunotoxins, there are ample potential applications for cytosolically delivered proteins that can be divided into those that (temporarily) add a function and those that block a pathway as inhibitors. Examples for the former include the delivery of enzymes [5] or proteins that reprogram or genetically modify cell populations in vivo [6,7]. The latter can be achieved either via direct protein inhibition or, as has attracted more attention recently, through enzymatic target modification, which, for instance, was achieved for RAS oncoproteins [8]. It can, in principle, also occur via targeted protein degradation [9], although in vivo targeted protein degradation using engineered proteins has not yet been reported. Blocking type applications can be especially powerful for cancer therapies or senescent cell removal, where blocking a pathway can drive cells that rely on this pathway for survival into apoptosis. Through binding of large interaction surfaces, proteins can easily mediate levels of specificity and modes of activity that are very difficult if not impossible to achieve with small molecules [10], thus massively expanding the druggable genome [11].

During the last years, there has been a steady development of approaches to deliver proteins into the cytosol of cells cultured in 2D in vitro systems [12,13,14,15]. However, there has been little success to translate these results into activity in vivo. Due to its challenges, access of therapeutic proteins to intracellular targets has been referred to as “high-hanging fruit”: highly desirable, but difficult to achieve [16].

This lack of progress may be attributed to the fact that in vivo protein delivery to cells that reside outside of the bloodstream presents several additional challenges. These challenges include extravasation in the organ of interest, adequate penetration into the tissue, and accumulation at the target cells [17]. For cytosolic delivery, as opposed to endosomal delivery, there additionally needs to be a moiety that enables the protein to escape endosomes; for instance, an endosomal escape peptide or a bacterial translocation domain [18]. Currently, there are several excellent computational models available that can be used to simulate the delivery of agents that bind to extracellular targets or to tumor tissues in general [19,20]. In contrast, in-depth modeling approaches to understand and define the requirements to yield effective cytosolic protein delivery for therapeutic applications have, to the best of our knowledge, not yet been reported.

Here, we present a mathematical model specifically built for the evaluation of protein engineering approaches directed at cytosolic protein delivery in in vivo solid tumor tissues. We employ this model to investigate how microvascular permeability, molecular size, affinity for a cellular target receptor, density of targeted receptors, and finally, mode of action, influence the drug activity. We focused our investigations on solid tumors, while reflecting on the consequences for protein-based therapeutics in various therapeutic areas. Our results demonstrate that for cytosolic protein delivery for a given area of application, in vivo activity can be rationally optimized through the proper tuning of parameters that are readily controllable through protein engineering and/or proper dosing regimes.

## 2. Materials and Methods

A technical explanation of the model, outcome parameters, as well as the rationale for the choice of input parameters and model assumptions is provided as a Supplementary Materials section.

## 3. Results

### 3.1. Modeling Protein Delivery

**Figure 1:**
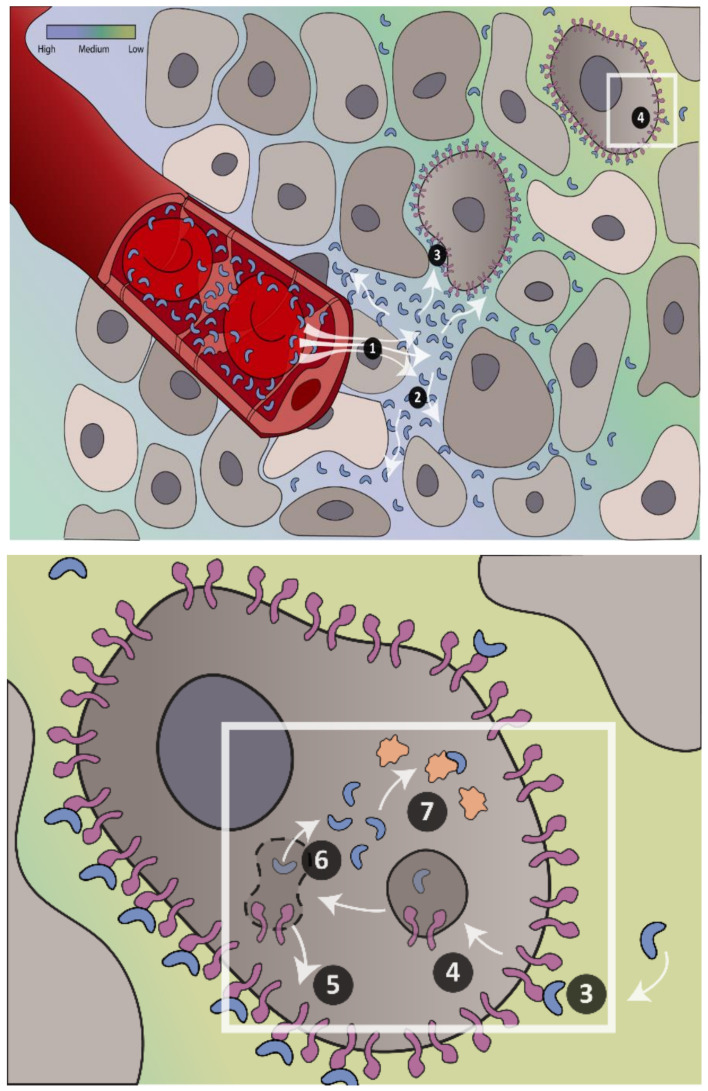
Schematic representation of protein delivery in vivo. Visualization of steps required for achieving cytosolic protein delivery in vivo in tumor tissue. (1) Extravasation; (2) transport in the interstitium; (3) binding to the cell surface; (4) internalization; (5) receptor recycling and synthesis; (6) endosomal escape; (7) binding of cytosolic target. The model contains six partial differential equations describing the changes in (i) the free therapeutic protein in the interstitium; (ii) the unbound cell surface receptor; (iii) the surface complex of receptor and therapeutic protein; (iv) the free therapeutic protein delivered to the cytosol; (v) the free cytosolic target protein; and finally, (vi) the inhibitory complex of therapeutic protein and cytosolic target. The bar in the upper-left corner shows the color scheme that represents the concentration of therapeutic protein delivered into the tissue. A complete mathematical description and a full explanation of its workings is given in the Supplementary Materials.

To generate a quantitative understanding of the requirements for effective in vivo cytosolic protein delivery, we developed a mathematical model incorporating key elements of cellular targeting, entry, and intracellular activity. The model was, in part, inspired by work from Thurber et al., in which antibody delivery in vivo was described within a Krogh geometry consisting of two concentric cylinders; the inner cylinder represented a capillary, and the outer represented the surrounding tissue (Figure 1) [19]. The model permits the in silico evaluation of all physical and biological phenomena that we reasoned would significantly influence cytosolic protein delivery and its therapeutic effects. Those parameters that relate to the engineered protein are size, affinity, intracellular binding, plasma half-life, half-life in the cytosol, as well as the effects of enzymatic target modification on the levels of functional cytosolic target proteins. Those parameters that relate more to the targeted tissue are vascular permeability, interstitial diffusivity, cellular internalization rates, and cytosolic delivery efficiency (Figure 1). An extensive description of how the model was designed and the rationale for the choice of all parameters is provided in the Supplementary Materials section. As a representative tissue, we focused on solid tumors of the breast overexpressing the tumor marker epithelial cell adhesion molecule (EpCAM), while in some instances, we used normal skeletal muscle tissue for comparison. We made a distinction between tumors that exhibited convection, i.e., those with functional lymph vessels, and those that did not, and therefore exhibited little or no fluid flow and where macromolecule transport is driven by diffusion. Results are, in most cases, described as the degree of inhibition as a function of time (i.e., inhibition of a cytosolic target protein), maximum inhibition (i.e., single time-point across the entire tissue), or as an inhibitory effect (effect integrated over time and entire tissue). A technical explanation of how these terms were calculated is given in the Supplementary Materials.

### 3.2. Simulation of Delivery and Therapeutic Effects In Vivo

**Figure 2:**
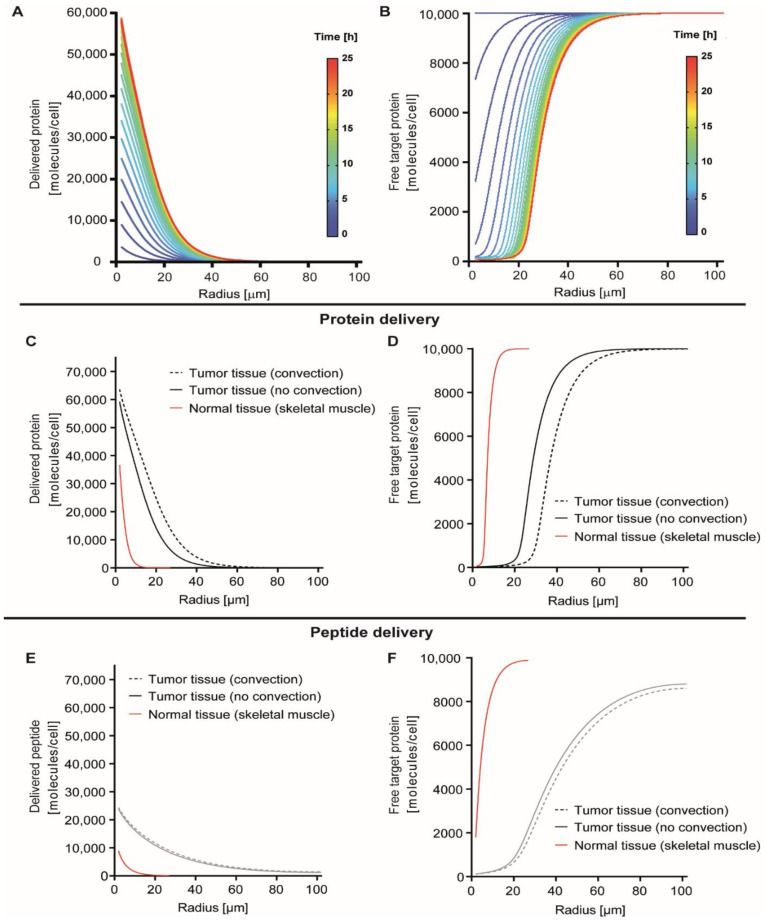
Radius of simulated delivery and biological effects of targeted agents in tumor tissue. (**A**) Simulation of radial and time-dependency of delivery of a model protein (70 kDa, target affinity: 68 pM) in tumor tissue without convection. Lines represent different timepoints shown up to the point of maximum delivery (24 h for protein). Supplementary Video S1 shows timepoints until 72 h for protein. We simulated a plasma half-life of 2.55 h, the half-life of the 83 kDa therapeutic enzyme laronidase [26], and modelled 5.3 × 10^5^ receptors/cell, as described for EpCAM on MCF7 cells [22]. (**B**) Depiction of the number of free cytosolic target proteins for the situation where an inhibitory target-binding protein is delivered under the same conditions as in (**A**). A total of 10,000 target molecules/cell were modelled, which is close to the mean level in eukaryotic cells as based on proteomic analyses [27]. Supplementary Figure S1 shows similar graphs as (**A**,**B**) for a model peptide. (**C**) 70 kDa model protein delivery (as in A) as a function of the distance from the lumen of the nearest blood vessel (radius) at the time-point of maximum delivery for distinct conditions. (**D**) Free target protein in tumor tissue compared to normal skeletal muscle tissue as a function of distance from the nearest blood vessel under conditions as in (**C**). (**E**,**F**). Same as for (**C**) and (**D**), but for a model 3.5 kDa targeted peptide. For the peptide, a half-life of 0.28 h was simulated, as reported for the therapeutic peptide calcitonin [25]. Time of maximum delivery for peptide is 17 h (vs. 24 h for protein). For detailed model assumptions, their rationale, and a technical explanation of the outcome terms on the *y*-axes, see the Supplementary Materials.

We initially simulated delivery of a protein with a molecular weight of 70 kDa binding to the tumor marker EpCAM. Binding properties were taken from the designed ankyrin repeat protein (DARPin) Ec1, which binds EpCAM with an affinity (K_d_) of 68 pM [21]. We assumed a tumor tissue containing a cell density of 2.9 × 10^8^ cells/mL, representing a tumor with a high cellularity, and a receptor density of 5.4 × 10^5^ receptors/cell, which we previously determined for EpCAM on MCF-7 cells [22]. As indicated above, we thus considered the tissue as a homogenous medium in which cellular structures are not explicitly defined. We simulated a starting protein plasma concentration of 1 μM, a concentration that can be realistically reached upon intravenous administration of a therapeutic protein [23] (Figure 2A). A sharp decline in the delivered protein concentration away from the capillary was observed, with the majority of protein being confined to the first 20 μm of tissue. When an EpCAM binder is combined with a moiety or vector that mediates efficient cytosolic delivery, e.g., an endosomal escape peptide or a bacterial toxin-derived translocation domain as we reported previously [24] (see Supplementary Table S2 for detailed information on assumptions), the simulated degree of target inhibition (see Supplements for a technical explanation) varies across different distances from the capillary (Figure 2B) and directly reflects protein delivery (Figure 2A), with maximum inhibition in the tissue reached at around 24 h (Supplementary Video S1).

We then evaluated the maximum cytosolic delivery and inhibition with a targeted protein (Figure 2C,D) or a targeted peptide (Figure 2E,F) in tumors with and without convection and compared it with delivery in normal tissue (skeletal muscle was chosen as a representative tissue because of the availability of experimental values of needed parameters in the literature). The relevant difference between both types of compounds is size, and we modelled a molecular weight (MW) of 3.5 kDa for the peptide, which is the MW of the calcitonin peptide hormone. Calcitonin is a medically used peptide for which half-lives have been determined in vivo in humans [25] (Supplementary Table S3). Employing a targeted peptide resulted in more rapid clearance but also faster penetration due to a higher interstitial diffusivity. We also included peptides because for this class of compounds, several strategies for cytosolic delivery have been described. For both proteins and peptides, cytosolic delivery and inhibition in tumors vastly surpassed that achieved in muscle tissue in terms of tissue reached, mostly due to the enhanced permeability of leaky blood vessels. Convection additionally contributed substantially to an enhanced depth of penetration for proteins (Figure 2C).

While targeted proteins were delivered in high concentrations to the tissue immediately adjacent to the capillary but rapidly decreased in concentration further away, peptides permeated the entire tissue more evenly by comparison, although in lower levels (Figure 2C,E).

### 3.3. Effect of Receptor Affinity on Peptide and Protein Delivery

**Figure 3:**
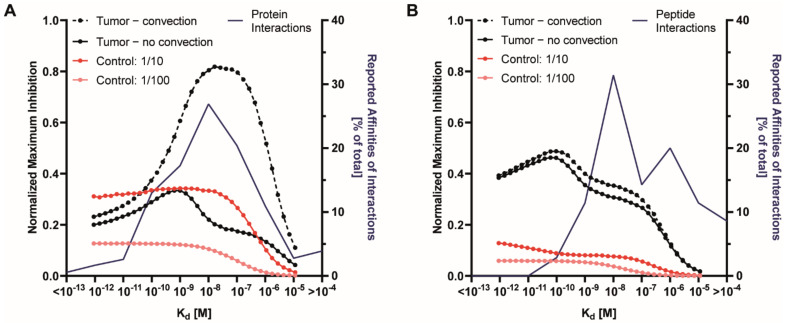
The dependency of delivery of proteins and peptides to tumors on receptor affinity. (**A**) The effect of affinity on inhibition by a targeted protein (70 kDa) (left *y*-axis) for tumor tissues with 5.3 × 10^5^ receptors per cell, and for a healthy control tissue (skeletal muscle), with 10× (1/10) and 100× (1/100) lower receptor levels. Dots identify the datapoints corresponding to individual simulations. The right *y*-axis shows a histogram of the relative frequency of protein interactions in the KOFFI database with their affinities per order of magnitude [30]. The KOFFI database collects binding kinetics data from biomolecular interactions from the literature. (**B**) Same as (**A**), but for targeted peptides.

The binding-site barrier is a phenomenon where high-affinity targeted agents bind tightly to receptors in the first cell layers encountered and only travel further into the tissue upon receptor saturation. The affinity for receptors, the receptor density, and the receptor internalization rate are well-established factors with respect to the binding-site barrier [19,28,29], although quantitative insights into the impact of the binding-site barrier on tissue penetration under different conditions are lacking. One approach to minimize the negative consequences of the binding-site barrier is to modulate the affinity of a targeted agent towards its cellular receptor. Importantly, a lower affinity may benefit tissue penetration. First, we determined the optimum receptor affinity for tumor targeting with or without convection for a fixed receptor density for both targeted proteins and peptides (Figure 3), as determined by maximal delivery to the tissue. Maximal delivery was defined as the situation when the average concentration across the tissue of the free target protein was at its lowest.

Convection facilitates the rapid transport of macromolecules from the immediate proximity of the endothelium deeper into the tissue, and its effect on protein delivery was therefore of particular interest. We found that higher affinities for targeted proteins were needed for the optimal delivery in tumors without convection. The presence of convection in tumors greatly reduced the optimal affinity and enhanced the overall therapeutic effect (Figure 3A).

For peptide delivery, on the other hand, almost identical optimal affinities were observed in the presence or absence of convection, and delivery only mildly increased in the presence of convection. When comparing peptides vs. proteins, peptides produced superior delivery in tumors without convection due to the higher permeability and diffusivity, while proteins surpass peptides in tumors with convection due to their longer plasma half-life (see also Figure 2C,E). The steeper decline in delivered molecules observed for proteins vs. peptides indicates a more pronounced binding-site barrier for slower-diffusing proteins.

Targeted proteins can easily be engineered to be highly selective and have affinities that are considered optimal by our modeling approach (Figure 3A). However, for peptides, the ability to obtain high affinities appears to be a limiting factor for their efficacy (Figure 3B), although peptides with very high affinities have been reported [30], including peptides that bind with subnanomolar K_d_ values to vascular endothelial growth factor (VEGF) receptor 2 and c-MET, or hapten peptides that bind to single chain variable fragments with affinities as high as 2.3 nM [31]. Interestingly, for targeted proteins, the optimal affinities for targeting tumors with and without convection, of 16.4 nM and 625 pM, respectively, for these simulated parameters, appear to be readily achievable, as demonstrated by the superimposition of the frequency distribution of protein affinities reported in the literature and compiled in the kinetics database KOFFI [30].

When investigating the effect of receptor density for our default conditions (i.e., using a proteinaceous EpCAM binder with an affinity of 68 pM), we noted a very strong dependency of the therapeutic effect on the receptor density (Supplementary Figure S3). This mirrors the effects of modulating receptor affinity (see Figure 3A) and reflects the characteristics of the binding-site barrier. Therapeutic effects were highest at around 1–2 × 10^5^ receptors/cell (Supplementary Figure S3).

### 3.4. Interplay Between Receptor Affinity, Receptor Density and Internalization Rate

**Figure 4:**
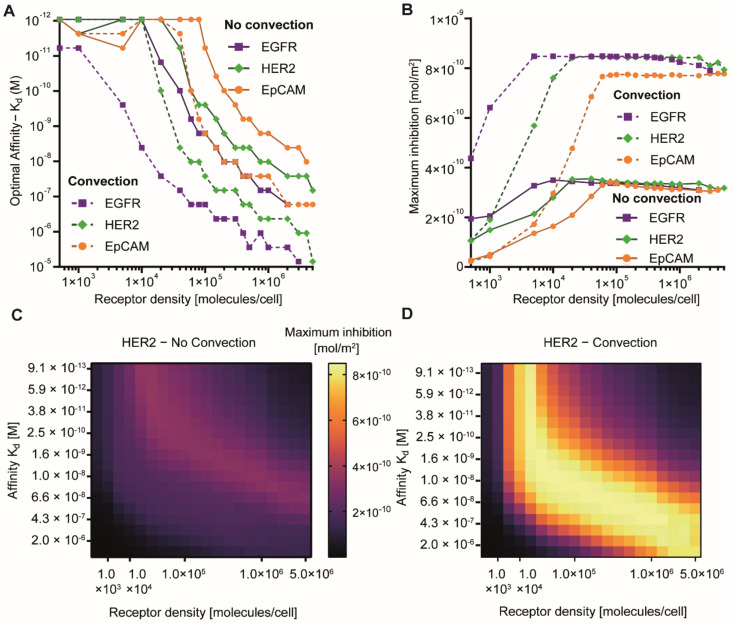
Optimal receptor affinities for overcoming the binding-site barrier. (**A**) Optimal receptor affinity in terms of overall delivery as a function of receptor density in tumors containing convection and those that do not for three reported rates of receptor internalization (k_e_). EGFR: ke = 0.08 min^−1^ [32]; HER2: 0.01 min^−1^ [32]; EpCAM: 0.002 min^−1^ [33]. Of note, internalization rates can differ substantially as a function of the ligand used for targeting. (**B**) Maximum inhibition achieved by optimizing receptor affinity for different receptor densities in tumor. Data correspond to datapoints seen in Figure 4A. (**C**) Heatmap showing the maximum inhibition produced by targeting a receptor with a reported internalization rate of HER2, at widely different expression levels, with targeted agents of varying affinities, in a tumor lacking convection. (**D**) Same as (**C**), but in a tumor with convection. Heatmaps for EpCAM and EGFR are shown in Supplementary Figure S4. For detailed model assumptions and a technical explanation of the outcome terms on the *y*-axes, see the Supplementary Materials.

At present, we are not aware of reports that relate the optimal affinity to receptor density and the receptor internalization rate. Ultimately, this knowledge may guide the choice of suitable receptors for targeting in the development of cancer therapies. Next to target affinity and receptor density, the internalization rate of cellular receptors plays a prominent role in determining the properties of the binding-site barrier [29]. This is illustrated by determining the affinity that yields the greatest therapeutic effect (i.e., optimum affinity) as a function of receptor density for receptors that differ in internalization rates, either in the presence or absence of convection (Figure 4A). Although internalization, recycling rates, and even trafficking routes for receptors can differ as a function of the ligand utilized for targeting (e.g., natural ligands vs. engineered binders), as well as the epitope addressed on a specific receptor, our simulations indicated that receptors which generally internalize faster, e.g., epidermal growth factor receptor (EGFR), necessitate a lower optimal affinity for maximal tissue delivery. Again, convection decreased the optimal affinity for delivery for all internalization rates and also increased the internalization rate-dependent differences (Figure 4). In our model, we simulated steady state levels of receptors by matching the rate of internalization with the rate of recycling, thus mimicking the delivery of non-toxic proteins (for details, see the Supplementary Materials). A fraction of targeted agents was delivered to the cytosol in the model, with the rest being degraded. Even in the presence of a moiety that mediates endosomal escape, degradation in the endolysosomal system is a common fate of endocytosed proteins, and only a fraction of internalized protein reaches the cytosol [18]. Remarkably, for a binder exhibiting the optimal affinity, the same overall magnitude of delivery and inhibition can be achieved largely irrespective of the receptor density and internalization rate (Figure 4B). This implies that targeting should not only focus on those receptors which are present at very high levels and for which very high affinity binders are available, which would increase the options for productive targeted drug delivery.

Nonetheless, specificity and potential off-target effects should always be taken into consideration. Notably, for diffusion-based delivery, maximal delivery is always lower, emphasizing the relevance of convection in targeted delivery to tumors.

### 3.5. Effect of Cold Dosing, Targeted Protein Degradation and Degradation-Resistant Proteins on the Binding-Site Barrier

As illustrated by our analyses so far, protein targeting deep into tissues faces several dilemmas. Small size, on the one hand, favors penetration, but on the other hand, leads to faster clearance. A high receptor affinity promotes effective cellular targeting, although limits penetration due the rapid capture of extravasated proteins by the first layer of cells, also referred to as the binding-site barrier. As an approach to overcome the binding-site barrier, cold dosing, where the therapeutic moiety is only present on a fraction of administered targeted agents, has been investigated for a long time. Mostly, this has been performed in the context of radiolabeled antibody [34,35] but, more recently, also in the context of antibody–drug conjugates [36,37]. In addition, there are multiple protein engineering approaches aimed to enhance intracellular delivery and/or subsequent therapeutic effects, some of which are only recently coming to the fore for engineered proteins. The therapeutic potency can be increased without affecting delivery itself, through either delivery of (target protein-inactivating) enzymes [8,38], targeted protein degradation [39], or by enhancing the stability of the delivered agent against proteasomal degradation [40,41].

**Figure 5:**
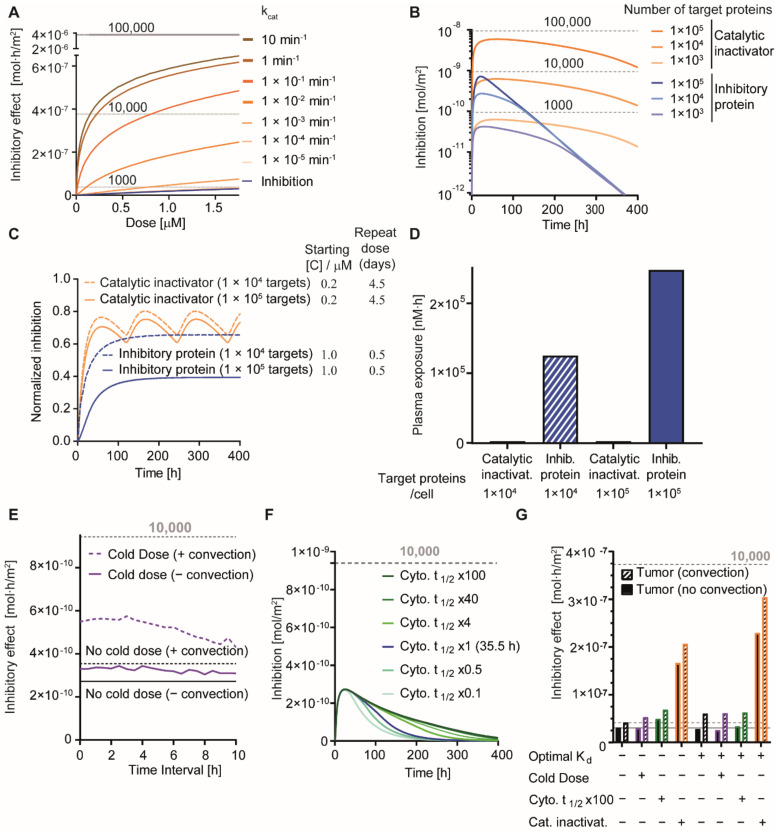
Strategies to overcome the binding-site barrier and prolong target inhibition in tumor tissue. (**A**) Simulation of therapeutic effects of a single dose, yielding an initial plasma concentration given on the *x*-axis of a targeted protein with an enzymatic cargo with different catalytic rate constants. The level of target protein in the cytosol was simulated to be 1 × 10^5^ per cell. (**B**) Effect over time for a dose corresponding to an initial plasma concentration of 1 μM of inhibitory binding proteins or catalytic inactivators for different levels of target protein present in the cytosol. (**C**) Effect of dosing regimens for binders and catalytic inactivators for 1 × 10^4^ and 1 × 10^5^ target molecules/cell. (**D**) Overall plasma exposure for the dosing regimens in (**C**). (**E**) Effect of cold dose on inhibitory effects in tumors containing convection and those that do not. (**F**) Effect of varying the half-life of delivered proteins in the cytosol on the inhibition. (**G**) Inhibitory effect of a single dose yielding an initial plasma concentration of 1 μM in tumors with/without convection. In the case of cold dosing: the cold dose had the same plasma concentration profile as the warm dose but no therapeutic cargo and a reduced size (10 kDa). Default conditions as in Figure 2A were used, unless specified otherwise. Cyto t_1/2_ × 100 indicates a 100× longer half-life in the cytosol of the targeted agent. For detailed model assumptions and a technical explanation of the outcome terms on the *y*-axes, see the Supplementary Materials.

In our simulations, catalytic inactivators represent both enzymes that inactivate target proteins and protein-based agents that induce target protein degradation, because for both types of activities, the functional outcome is the same. Given our assumptions (see Supplementary Materials), catalytic agents strongly outperform binders whose effect is limited to inhibition by direct blockade of the protein—protein interaction, in particular at lower concentrations (Figure 5A) or when the target protein concentration is high (Figure 5B). For instance, at a target protein level of 1.0 × 10^5^ molecules/cell and using catalytic inactivators, a very strong and pronounced inhibition can be observed as compared to inhibition by binding only (Figure 5B). Irrespective of the presence of a binding-site barrier, the high activity upon cytosolic delivery of even small amounts of catalytic inactivators results in much stronger therapeutic effects throughout the tissue (Figure 5A,B). Assuming identical delivery properties and stabilities in the cytosol, the effects are much longer-lasting, because after removal of the inactivator, the pool of target proteins first has to be replenished by translation (Figure 5B). Even with relatively low estimates of rates of inactivation (low k_cat_ values), the effects are remarkably potent (Figure 5A).

Enhanced effects of catalytic cargoes can be further increased by repeated dosing approaches. For a scenario with application of a dose resulting in a 200 nM concentration of an enzymatic cargo in the plasma approximately every 4.5 days, greater therapeutic effects are produced as compared to the twice-daily administration of binders to a plasma concentration of 1 μM (Figure 5C). In a clinical setting, the lower plasma exposure required for catalytic agents (Figure 5D) might have considerable benefits with respect to eliciting non-specific side effects as well as immunogenic reactions [42]. We subsequently investigated the effect of cold dosing and analyzed the influence of the time interval between warm and cold dose in tumors exhibiting convection and those that do not. Cold dosing enhanced the overall inhibitory (i.e., therapeutic) effect, with stronger effects in tumors exhibiting convection and in the presence of shorter time intervals (Figure 5E). Of note is that for binders that show optimal receptor affinities, cold dosing tends to have little effect (see below; Figure 5G).

As alluded to before, an alternative approach to increase the activity of proteins delivered to the cytosol is to enhance the stability against degradation, for instance, by removing lysine residues on the protein surface, through which the canonical pathway of ubiquitination and proteasomal degradation occurs [43]. Alternatively, the (partial) use of D-amino acids can improve stability [40,41], because protein stretches composed of D-amino acids are resistant to proteolytic degradation. A higher cytosolic stability was simulated as a longer cytosolic half-life of binders in our model (Figure 5F). Our findings indicate that a moderate enhancement of the cytosolic half-life (4×) can make the therapeutic effect much longer lasting. By contrast, a large increase in half-lives (40×/100×) shows limited additional effects due to the constant synthesis of new target proteins. Conversely, shortening the half-life cuts down the duration of the therapeutic effect, although the maximum level of inhibition (i.e., at a single time-point) is surprisingly unaffected even when a 10-fold shorter cytosolic half-life is assumed (Figure 5F).

When comparing strategies side-by-side, an optimization of receptor affinity was more powerful than cold dosing with respect to maximizing effects throughout the tissue (Figure 5G). When investigating the maximum inhibition instead of the inhibitory effect achieved by these different strategies, the efficacy of a cold dose in tumors with convection is strong when the affinity is not optimal, but has no positive effects when an optimal K_d_ is modelled (Supplementary Figure S5). Extending the cytosolic half-life is, within the conditions tested, ineffective with respect to increasing the level of maximum inhibition.

## 4. Discussion

The ability to deliver proteins efficiently into the cytosol of specific cells in vivo would enable numerous novel therapeutic opportunities [16], including the interference with signaling pathways in cancer and senescent cells, the restoration of missing functions in genetic diseases, and potentially even the ability to reprogram cells in vivo to restore or redirect their identity [44]. Here, we built a mathematical model for investigating the challenges of protein delivery in vivo and to evaluate the merit of engineering approaches that are geared towards overcoming these challenges. As we demonstrate, the results provide clear guidelines for protein engineers on how to design proteins more rationally towards specific applications. While our quantitative analyses are focused on cancer targeting in vivo, the findings are also qualitatively pertinent to other disease areas. Furthermore, variants of the model can be employed to study protein transport and activity in microfluidic models mimicking the tumor microenvironment with various degrees of complexity, and we are actively pursuing this line of research.

As a starting point, it is useful to consider that approaches that report cytosolic protein delivery in 2D systems in vitro often report values of cytosolic concentrations for the delivered protein that are comparatively high (mid- or high-nanomolar range) [18,24,45,46,47,48,49,50,51,52,53,54], which is well over the average level of molecules of a specific protein in a cell, which is around 2000–8000 molecules/cell (~2–10 nM) [27]. Hence, addressing cytosolic targets in vitro is already possible for some applications. However, similarly to challenges associated with a homogenous delivery to targeted agents to extracellular receptors [55], delivery challenges and a rapid degradation in the cytosol imply that reaching these levels and concomitant biological effects in vivo remains very difficult.

The binding-site barrier is an often-mentioned factor that limits effective tissue penetration [28,56]. While it has long been known that reducing the affinity of a binder for a specific receptor may facilitate tumor penetration [57], our simulations indicate that tuning the receptor affinity for a particular receptor density, receptor internalization rate, and the presence or absence of convection can be very powerful in increasing targeting. For our default scenario (agent binding EpCAM with 68 pM), we observed a 16% increase in maximum inhibition in tumors without convection and a 118% increase in tumors with convection. The 16% increase in tumors without convection is modest because our starting affinity was fairly close to the optimal affinity already (which was 625 pM), although in general, improvements in tissue delivery by optimizing affinity surpass those achieved by cold dosing approaches. Two notable outcomes of our simulations are (i) that the optimal affinity for delivery is much lower in tumor tissue exhibiting convection compared to its counterpart without convection, with optimal affinities differing between one and three orders of magnitude, depending on the receptor internalization rate and receptor density (Figure 4); and (ii) that within a large range of receptor internalization rates and receptor densities, equal overall therapeutic effects can be accomplished, provided the affinity for the receptor has been optimized (Figure 4).

With contemporary screening and protein engineering approaches, the tuning of affinities is a feasible undertaking. Nevertheless, in practice, a balance needs to be found between the optimal affinity for a specific set of conditions (e.g., type of receptor, receptor density, convection) and the degree of heterogeneity in the tumor—often, tumors are characterized by regions that differ in extracellular matrix (ECM) densities, in the presence of convective flow [58,59], and in levels of receptor expression. As a consequence, heterogeneous delivery is often observed in antibody-treated tumors [60], and this heterogeneity is even more pronounced when utilizing antibody–drug conjugates that already have dose-limiting toxicities at low doses [36]. Our analyses on the interplay between the various factors that govern cellular delivery (receptor density, internalization, affinity, convection) will aid the understanding of the impact of heterogeneity on delivery. While tumor heterogeneity is an inherent and challenging aspect of tumor targeting that complicates finding one optimal solution, by covering broad ranges of values in our simulations, the decision to address a receptor with higher or lower affinity can be made more rational.

We focused our investigations on the binding and internalization of low- to medium-sized proteins that reach the target cells through active targeting. For that reason, our model does not apply to delivery by the enhanced permeability and retention (EPR) effect, which describes the passive accumulation of large entities, e.g., nanoparticles, in tumor tissue due to a high local microvascular permeability and a poor lymphatic clearance [61]. However, for delivery by the EPR effect, the incorporation of modalities for active targeting has only limited added value [62].

Our analyses demonstrate the promise of two strategies to mitigate the effect of the binding-site barrier: optimizing the affinity towards the cell surface receptor and enzymatic target modulation or targeted protein degradation (Figure 5G).

Optimizing the affinity towards cell surface receptors was identified as an approach that could facilitate overcoming the binding-site barrier in a straightforward manner. Importantly, affinity optimization is not synonymous with achieving as high an affinity as possible. The findings hold for both tumors exhibiting convection and those that do not.

Cytosolic delivery of enzymes that inactivate oncogenes was recently demonstrated through the diphtheria toxin-mediated delivery of an enzyme that inactivates wild-type and mutant RAS [8]. The k_cat_ of the RAS-cleaving enzyme that was used was 2.35 min^−1^ [63], and is on the high end of the range of catalytic rates that were simulated by us (Figure 5A), emphasizing that this particular approach is indeed very powerful. While tumor growth inhibition in vivo was achieved, challenges associated with full tumor penetration were identified that contributed to suboptimal therapeutic effects. Targeted protein degradation, as a more generic approach for intracellular protein depletion, also exhibits great potency and is rapidly moving towards clinical application. Although current agents to trigger targeted protein degradation are mostly small molecule-based rather than protein-based [64,65,66], a monobody binder targeting the Lck tyrosine kinase fused to von Hippel-Lindau (VHL), a substrate receptor of the Cullin2-E3 ubiquitin ligase complex, has recently been shown to mediate targeted protein degradation in vitro upon cytosolic delivery [39]. Notwithstanding its potential, the high potency of targeted protein degradation or enzymatic inactivation means that off-target delivery could prove more toxic at lower concentrations (see Figure 5A,B), which emphasizes the necessity to place great importance on the specificity of the targeting approach.

A better quantitative understanding of in vivo protein delivery has the potential to act synergistically with a better quantitative knowledge of how many proteins are necessary to exert a specific effect. This can range from targeted protein degradation of highly overexpressed anti-apoptotic proteins in cancer cells or senescent cells, e.g., members of the BCL-2 protein family [67,68], to the delivery of transcription factors over several days for in vivo reprogramming efforts, as was recently accomplished using an mRNA-based approach [69]. Given the immunogenic risks associated with long-term therapy, application areas beyond cancer in which transient administration is sufficient are particularly promising, especially those where partial effectivity already gives rise to substantial therapeutic effect. For example, by interfering with signaling pathways that mediate the survival of senescent cells, a short-term protein-based approach to interfere with these pathways may remove a fraction of the senescent cells, which is already expected to yield multiple health-related benefits [70].

### Limitations and Future Perspectives

A limitation in our model is that surface charge of the delivered proteins has not been taken into account. Surface charge has been shown to affect both the permeability and interstitial diffusivity of macromolecules and nanoparticles. and this effect has been attributed to interactions with the negatively charged basement membranes and ECM, respectively [71,72,73]. Our reliance on data from neutral (i.e., non-charged) dextrans for some parameters, for reasons of availability, means that the extrapolation towards proteins with very different charge characteristics has to be performed with caution. However, upon the availability of better-suited data for proteins with specific charges, these data can be easily implemented in our model.

For our analyses, we chose a shorter plasma half-life for the therapeutic proteins than what is common for IgG antibodies, because for cytosolic delivery, full-length antibodies are an unlikely choice and antibodies also show only poor tissue penetration [16]. Nevertheless, for non-IgG proteins, plasma half-lives can also now be engineered to a large extent; for example, through the inclusion of Fc segments [74] or by fusion to albumin-binding domains to exploit FcRn receptors [75,76]. The presented model has been employed here to extract general principles but should be refined towards protein engineering approaches for specific applications. This can be achieved through the implementation of more detailed and context-relevant experimental data obtained from in vivo studies and studies with microfluidic models. Experimental studies can refine the model and provide better estimates of plasma and intracellular half-life, the approach utilized for mediating endosomal escape, interstitial diffusivities, surface receptor internalization as a function of the targeting agent, ubiquitination rates, and microvascular permeabilities. When these values are unknown, a broad range of values can be tested to investigate the sensitivity towards there parameters. This would help to understand the relevance of measuring and/or optimizing these values.

At present, the model also does not recapitulate the complexity of tumor vasculature in vivo. However, because the aim of the model is to understand extravasation from the vasculature, tissue penetration, and entry into cells in the direct vicinity of a capillary, we do not consider this simplification a significant limitation. Finally, through incorporating specific rates of transcytosis through receptors such as transferrin or insulin receptors, which are often used for delivery across the blood–brain barrier [77,78], our model can also be employed to simulate delivery to the brain or other tissues in which transcytosis is the main mode of transport.

In conclusion, we have shown that the “high-hanging fruit” of proteins that exert their therapeutic effect inside cells is well within reach of the protein engineering approaches that are currently being pursued. Nevertheless, the proper engineering of their characteristics will be crucial, and models such as the one presented here will guide this way.

## Data Availability

Data are available for reuse upon reasonable request.

## Supplementary Materials

The following are available online at https://www.mdpi.com/article/10.3390/pharmaceutics13040562/s1, Supplementary Materials, Figure S1: Radius of simulated delivery and biological effect of a targeted peptide; Figure S2: Derivation of k_on_ and k_off_ rates utilized for varying the affinity in simulations; Figure S3: Effect of receptor density on target protein inhibition; Figure S4: Heatmaps showing the maximum inhibition achieved by targeting receptors; Figure S5: Comparison of the maximum inhibition produced by different strategies. Table S1: Time-dependent partial differential equations of model; Table S2: Default parameters (tumor tissue/70 kDa protein); Table S3: Specific parameters for tissue and molecular weight dependence; Table S4: Modified partial differential equations used to model catalytic degradation; Table S5: Partial differential equations used to model cold dosing. Supplementary Video S1: Time-lapse of maximum inhibition.

## Abbreviations

DARPin, designed ankyrin repeat protein; ECM, extracellular matrix; EGFR, epidermal growth factor receptor; EpCAM, epithelial cell adhesion molecule; EPR, enhanced permeability and retention; VEGF, vascular endothelial growth factor; VHL, von Hippel-Lindau.
