# Supplementary material for: A Computational Investigation of In Vivo Cytosolic Protein Delivery for Cancer Therapy
Source: Pharmaceutics. 2021 Apr 15;13(4):562. doi: 10.3390/pharmaceutics13040562 (PMC8071550; doi:10.3390/pharmaceutics13040562)
Supplement: Supplementary file 1 [file pharmaceutics-13-00562-s001.zip › pharmaceutics-1159455-SI.pdf]

pharmaceutics

MDPI

Supplementary Materials: A Computational Investigation of In Vivo Cytosolic Protein Delivery for Cancer Therapy

Camilo Torres, Simon Dumas, Valentina Palacio-Castañeda, Stéphanie Descroix, Roland Brock and Wouter P.R. Verdurmen

List of Symbols

Variables	Unit	Definition
D	cm²/s	Diffusion constant of targeted agent
D_2	cm²/s	Diffusion constant of cold dose
V_{int} / V_{total}	dimensionless fraction	Fraction of interstitial volume
V_{cell}	L	Cell volume
Re	molecules/cell	Number of cell surface receptors per cell
k_1	h^{-1}	Plasma clearance constant
k_{on}	$M^{-1}s^{-1}$	Surface receptor on-rate
k_{con}	$M^{-1}s^{-1}$	Surface receptor on-rate (cold dose)
k_{off}	s^{-1}	Surface receptor off-rate
k_{coff}	s^{-1}	Surface receptor off-rate (cold dose)
k_e	s^{-1}	Internalization rate
Cde	dimensionless fraction	Cytosolic delivery efficiency
k_{off2}	s^{-1}	Intracellular off-rate
k_3	s^{-1}	Intracellular degradation rate of targeted agent
k_4	s^{-1}	Intracellular degradation rate of target protein
k_{on2}	$M^{-1}s^{-1}$	Intracellular on-rate
k_5	s^{-1}	Intracellular complex degradation rate
T	molecules/cell	Number of target proteins per cell
TA	M	Concentration of therapeutic agent
B	M	Complex between TA and Re
D	M	Complex between cold dose and Re
I	complexes/cell	Inhibitory complex of blocking agent and target
TAcyto	molecules/cell	Therapeutic agent concentration in cytosol
T_0	molecules/cell	Target protein level at t = 0
ε	dimensionless fraction	Void fraction/available volume fraction for interstitial diffusion
$\upsilon_{int\,t}$	µm/s	Velocity of interstitial flow
P	m/s	Microvascular permeability
C	M	Concentration of cold dose protein

Publisher's Note: MDPI stays neutral with regard to jurisdictional claims in published maps and institutional affiliations.

Copyright: © 2020 by the authors. Submitted for possible open access publication under the terms and conditions of the Creative Commons Attribution (CC BY) license (http://creativecommons.org/licenses/by/4.0/).

SUPPLEMENTARY METHODS

1. Design of the Mathematical Model

A mathematical model was built which permits the evaluation of the importance of parameters related to the therapeutic or targeted agent (TA, protein that binds a cell surface receptor) as well as the targeted tissue. The model was designed in part by building upon work from Thurber et al. regarding a model with cylindrical geometry (Krogh-cylinder) for simulating antibody delivery in vivo. This model treats the tissue as a homogenous medium in which cellular structures are not explicitly defined [1]. The model was built with COMSOL Multiphysics as a set of partial differential equations, with terms and boundary conditions incorporating those physical and biological phenomena that we reasoned would significantly influence cytosolic protein delivery and its therapeutic effects.

For the Krogh cylinder, a narrow cylinder, representing a capillary, is encased by a larger cylinder, which constitutes the surrounding tissue. The wall of this inner cylinder is the endothelium, across which solute exchange occurs. The radius of the inner cylinder was chosen to be 2 µm, a value that represents a normal radius of capillaries found in the body [2]. The radius of the larger cylinder is determined by the diffusion limit of oxygen in tissues, which is approximately 100 µm [3]. Beyond this distance, insufficient oxygen

can be supplied to sustain cells, which can give rise to necrosis, as seen in many types of cancers [4].

This cylindrical geometry is compressed to a single dimension in the model considering a radial symmetry. A single line represents the radius of the entire cylinder, with the inner lumen of the capillary found at r = 0–2 μm, the endothelial barrier at r = 2 μm, and the tissue at r = 2–102 μm. When convection is modelled, clearance by the lymphatic system is implicitly modelled at the furthest edge of tissue, at r = 102 μm, being equivalent to the degree of convection in the tissue. No lymphatic clearance was present in the convectionless implementations of the model.

2. In-Depth Explanation of Equations

The partial differential equations compiled in Supplementary Table 1 (Equation 1-6) describe the transport of the targeted agent in the tissue interstitium, interactions with the cell surface receptors, and the formation of the cell surface-bound complex (see Equation (1), Equation (2), and Equation (3), respectively), as well as the internalization and translocation of the targeted agent into the cytosol. Additionally, interactions of the targeted agent with its target protein in the cytosol, and the formation of an inhibitory complex are described (see Equation (4), Equation (5), and Equation (6), respectively). Plasma clearance of the targeted agent and the extravasation of the targeted agent were modelled as boundary conditions at r = 2 μm, whereas the equations of Supplementary Table 1 are only valid for r = to 2–102 μm. The concentration of a targeted agent in the capillary lumen was modelled to decay in a mono-exponential fashion according to the formula:

$$TA_{plasma}(t) = TA_0\ e^{-k_1 t} \tag{7}$$

where TA_0 (M) is the initial concentration of the targeted agent in the capillary (0 < r < 2 μm), and $k_1 = \frac{\ln(2)}{t_{\frac{1}{2}}}$, with $t_{\frac{1}{2}}$ (h) being the plasma half-life of the targeted agent.

The extravasation of the targeted agent across the endothelium is modelled by the solute flux formula:

$$J_{TA} = -\ P_{app.}\delta TA \tag{8}$$

where $P_{app.}$ (cm/s) is the apparent endothelial permeability of the targeted agent. It was measured experimentally, and thus includes the effect of solvent drag caused by convection. Here, δTA is the difference in concentration, across the endothelium.

As in Thurber et al. [1], we implemented a mixed boundary condition where both value and derivative are involved. ε is added in the boundary condition to take into account the tumor porosity that decreases the apparent concentration in the tumor.

$$J_{TA} = -P_{app}(\varepsilon TA_{plasma} - TA_{r=2\mu m}) \tag{9}$$

In the extravascular space, the diffusion of the targeted agent in the matrix and the interstitial flow from the capillary, the contribution of convection, are modelled by the first two terms of Equation (1) (D (cm^2/s) and u_{int} (μm/s)), which are the interstitial diffusion coefficient of the targeted agent and the interstitial flow velocity, respectively.

The interactions between the targeted agent and cellular receptors are modelled by considering the extravascular space to be a homogenous solution of cell receptors, Re, which is a geometric simplification from reality where receptors are locally clustered on cells or even on specific membrane domains on cells. Key properties such as overall density, affinity, and internalization rate are appropriately modeled; therefore, we consider this an acceptable simplification. Furthermore, Thurber et al. found this approach to yield simulated data in line with experimental findings [5]. The initial and steady-state molar concentrations of this solution are calculated from the receptor density by the following equation:

$$Re_0\ (M) = \frac{Re_0\ \left(\frac{molecules}{cell}\right)}{\mathrm{V_{cell}}N_A}(1 - \frac{\mathrm{V_{int.}}}{\mathrm{V_{total}}}) \quad (10)$$

where $\mathrm{V_{cell}}$ is the average volume of a cell, and $\frac{\mathrm{V_{int.}}}{\mathrm{V_{total}}}$ is the fraction of interstitial volume to the total extravascular volume, with $\mathrm{V_{total}} = \mathrm{V_{int.}} + \mathrm{V_{all\ cells}}$. Note that the void fraction (ε) is smaller than the fraction $\frac{\mathrm{V_{int.}}}{\mathrm{V_{total}}}$ due to accessibility restrictions in small spaces that depend on the properties of the extracellular matrix. Hence, ε is also referred to as the available volume fraction. Values were taken from the literature (Supplementary Table 2).

The synthesis of new receptors and the recycling of these receptors is modelled by the first and last terms of Equation (2). k_e is the internalization or endocytic rate constant, and when $Re = Re_0$, these terms cancel out to produce the steady state receptor concentration.

The binding and unbinding reactions of the targeted agent to the receptors are modelled according to standard binding kinetics, with on- and off-rates:

$$TA + Re \underset{k_{off}}{\overset{k_{on}}{\rightleftharpoons}} B \quad (11)$$

Terms containing k_{on} and k_{off} in Equation (1), Equation (2), and Equation (3) describe the binding of the targeted agent to the receptors; thus, the formation of the complex B, and also the dissociation of this complex. Similar terms, containing $k_{on\,2}$ and $k_{off\,2}$, are used to describe the formation and dissociation of the inhibitory complex I inside the cytosol (see Equation (4), Equation (5), and Equation (6)). However, because the binding of the targeted agent to the receptors is limited to diffuse in the interstitial available volume only, the rate constant k_{on} is divided by ε, the fraction of available extravascular volume for the targeted agent, which is also known as the void fraction. The internalization of the targeted agent is modelled by the third term of Equation (3) (k_eB) and depends on the internalization rate constant k_e and the complex concentration B.

The translocation of the targeted agent from the endosome to the cytosol is in turn given by the first term of Equation (4). The cytosolic delivery efficiency, Cde, is the parameter that describes how efficiently this process occurs as a fraction, meaning that when Cde = 0.1, only 10 % of the endocytosed targeted agents reach the cytosol. Translocation occurs instantaneously in the model. The degradation of the targeted agent, as well as that of the inhibitory complex, are modelled with the rate constants k_3 and k_5, respectively. Similarly, the endogenous target protein is degraded according to its own degradation constant, k_4. Target protein synthesis is modelled in Equation (5), similarly to receptor synthesis, to give a steady state concentration equal to the initial concentration given by Equation (12).

$$T_0\ (M) = \frac{T_0\ \left(\frac{molecules}{cell}\right)}{\mathrm{V_{cell}}N_A} \quad (12)$$

3. Rationale for the Choice of Parameters

A realistic plasma concentration reached after intravenous injection of anti-cancer monoclonal antibodies was chosen to be the default initial plasma concentration [6]. Note that our default size, 70 kDa, is smaller than the ~150 kDa of a full-length IgG antibody.

The plasma half-life of Laronidase (2.55 h), an 83 kDa therapeutic enzyme, was used as the default half-life for this model [7]. Three other FDA-approved proteins, between 65 and 70 kDa, have very similar half-lives of 2–3 h: Aprotinin, Desirudin, and recombinant C1 esterase inhibitor [8]. Moreover, 80 kDa minibodies have plasma half-lives of 2.2–3.5 h [9].

The apparent microvascular permeability (P_{app}) and diffusion coefficient (D) were taken from studies for a 70 kDa dextran with xenografts in dorsal-skinfold chambers in mice [10] and in rabbit ear-chambers [11], respectively. The interstitial flow velocity, v_{int}, was measured with bovine serum albumin (BSA) by fluorescence photobleaching, also in a rabbit ear-chamber tumor model [12]. The available interstitial volume fraction or void fraction (ε) was experimentally measured in rat mammary tumors for IgG [13], and has been used in similar tumor models [14,15].

For the epithelial cell adhesion molecule (EpCAM) the receptor density, Re_0, in the MCF-7 cell line was used [16]. The k_{on} and k_{off} rate constants used for the binding of the cell surface receptors were those of Ec1, an EpCAM-binding DARPin [17]. The rate of internalization, k_e, was derived from flow cytometry data of an anti-EpCAM antibody uptake in SKOV-3 cells [18].

The median calculated half-life of the HeLa cell proteome was used as the half-life, $t_{1/2}$, of both the target protein T and the complex I [19]. The average cell volume, V_{cell}, of MCF-7 cells was used [20]. The interstitial fluid volume fraction, V_{int}/V_{total}, was measured with small molecule tracers ex vivo in rat fibrosarcoma xenograft tissue [21]. The steady-state target concentration of the target protein was close to the median concentration of all proteins determined by mass spectrometry proteome analysis in yeast [22].

The rate constants chosen for the binding of an intracellular target protein, k_{on2} and k_{off2}, were those determined for the DARPin 012_F12 against BCL-xL by surface plasmon resonance [17].

When normal tissue/skeletal muscle was modelled, a different tissue diameter was used to consider the reported intercapillary distances in skeletal muscle [23]; however, the capillary diameter was kept constant at 4 µm. Skeletal muscle was modelled with a 27 µm outer radius, resulting in 25 µm of "tissue radius". When default protein delivery was modelled in these tissues, the microvascular permeabilities and diffusivities were those given in Supplementary Table 3 for targeted agents with a molecular weight (MW) of 70 kDa. The same plasma half-life as in Supplementary Table 2 was taken for simulations.

4. Dependency of Delivery and Inhibition on Molecular Weight

The comparability of values was a priority as the parameters required for this analysis were compiled. Despite modelling proteins, in some instances we preferred to take values for dextrans, for which parameters are more consistently reported and which allowed us to avoid mixing too many different sources. Dextran permeabilities and diffusivities were taken from in vivo measurements in the respective tissues (Supplementary Table 3). For permeability values in skeletal muscle, we used an experimental value derived from inulin with a molecular weight of 5.5 kDa, and two other values were calculated from Peclet numbers and by varying hydraulic permeabilities in rat cremaster muscle [24]. The ε value for muscle seen in Supplementary Table 3 was calculated as follows:

$$\varepsilon(muscle) = \varepsilon\ (tumor) / \frac{V_{int.}}{V_{total}}(tumor)\ *\ \frac{V_{int.}}{V_{total}}(muscle) \tag{13}$$

For some simulations, the tumor was modelled with and without convection, reflecting distinct tumor tissues or distinct areas within tumor tissues [25]. Skeletal muscle was modelled with convection, using a value of 0.59 µm/s for interstitial velocity [12].

5. Dependence of Delivery and Inhibition on Receptor Affinity

For the experiments in which the affinity of the targeted agent towards its cell receptor was varied, we derived k_{on} and k_{off} values from Koffi, a binding kinetics database [26]. Specifically, we took 539 datapoints of protein and peptide interactions for affinities in the range of 10^{-5} to 10^{-12} and derived functions by linear regression describing the dependency of on- and off-rates on affinity: $Log_{10}(k_{on}) = -0.35 * K_D + 2.26$ (R^2 of linear regression: 0.30) / $Log_{10}(koff) = 0.66 * K_D + 2.38$ (R^2 of linear regression: 0.57) (Supplementary Figure 2).

6. Efficacy of Different Strategies to Improve Delivery and Inhibition

Targeted Protein Degradation: The modelling of catalytic degradation by targeted agents was implemented using classical enzyme kinetics:

$$TA_{cyto} + T \underset{k_{off2}}{\overset{k_{on2}}{\rightleftharpoons}} I \xrightarrow{k_{cat}} TA_{cyto} \tag{14}$$

The above equation was used to modify the partial differential equations (4) and (6) from Supplementary Table 1), yielding the equations used to model catalytic inactivators (Supplementary Table 4, Equation (15) and Equation (16)). Our approach does not take into account the complexity of the individual steps of targeted protein degradation including, but not limited to, the binding of the cellular machinery required for ubiquitination, ubiquitin substrate regulation, and possible saturation of proteasomal degradation [27].

The rate constant, k_{cat}, is analogous to the rate constant with which an E3 ligase causes the mono-ubiquitination of its target. As a simplification in our model, we assumed that this activity results in degradation of the target protein.

Catalysis rates of engineered catalytic inactivators have, to the best of our knowledge, not yet been reported; therefore, we employed reported catalytic rates of endogenous E3 ligases to their targets. Our default k_{cat} value was 0.93 min^{-1} for the ubiquitination of β-catenin by the UbcH5c (E2) and the $SCF^{\beta\text{-TrCP}}$ (E3) enzymes [28], although rates as high as 0.43 s^{-1} have been reported for ubiquitin transfer to a peptide derived from phosphorylated human cyclin E/CCNE1 [29].

Cytosolic Half-life Extension: The extension of the cytosolic half-life of the targeted delivery agent was accomplished by changing the degradation rate constant of the intracellular targeted agent, k_3, appropriately.

Cold Dosing: The cold dose was modelled by introducing two new partial differential equations (Supplementary Table 5): Equation (17) and Equation (19). Equation (17) describes the diffusion, convection and binding/release of the cold dose protein with the receptor. Equation (19) describes the evolution of the cold dose–receptor complex. Additional terms had to be added in Equation (2) to take into account the effect of cold dose on the free receptor; the modified equation is given in Equation (18). The time interval between cold and warm dose also had to be implemented. We used an equation similar to Equation (7) to describe the decay over time of the cold dose protein in plasma.

$$C(t) = C_0\, e^{-k_1 t} \tag{20}$$

where C_0 is the initial concentration of cold dose protein in plasma.

For the warm dose, we used the same decay function but shifted in time. This was implemented by using a step function to remove negative values:

$$TA(t) = TA_0\, e^{-k_1(t-t_{dose})} \times H(t-t_{dose}) \tag{21}$$

where $H(t)$ is the Heavyside function (step function), and t_{dose} is the timepoint at which the dose is delivered. A slight smoothing had to be added to the Heavyside function for numerical stability.

The vascular permeability and interstitial diffusivity of 10 kDa dextran was used to model the cold dose (Supplementary Table 3). However, the plasma half-life and starting plasma concentrations were left unchanged (see Supplementary Table 2).

Repeated dosing: Several doses were modelled in a similar manner to cold dosing. Several concentration decay functions were added and shifted at different delay times:

$$TA_{plasma}(t) = \sum_{n=1}^{N_{doses}} TA_0\, e^{-k_1(t-t^n_{dose})} \times H(t-t^n_{dose}) \tag{22}$$

where N_{doses} is the number of doses, and t_{dose}^{n} is the timepoint at which the nth dose is delivered.

7. Technical Explanation of Outcome Measures

The term "inhibition" is defined as the integral along the tissue radius, or area under the curve "AUC", of the steady state (initial) target protein concentration (T_0) minus the integral of the target protein concentration at that timepoint (T(t), i.e., inhibition = AUC(r) of T_0—T(t)). This value mirrors the integral of the inhibitory complex concentration in the case of binding inhibition ("AUC(r) of I") and facilitates the comparison to the degree of "inhibition" caused by, e.g., catalytic inactivators. This value changes over time; therefore, it can be used to visualize the time-dependency of the biological effect produced.

The "maximum timepoint" is the timepoint when the concentration of the free target protein is at its lowest on average across the tissue radius "AUC(r) of T_0—T_{max}", and, in the case of binding inhibition, that of the inhibitory complex is at its highest. This value is thus a measure of how deeply the targeted agent has penetrated into the tissue, and is strongly affected by properties of the binding-site barrier.

The term "inhibitory effect" is the integral over time of the "inhibition"; "AUC(r,t) of T_0—T(t)". This value, unlike the "maximum inhibition", takes the time factor into account and condenses this to a single value. It is affected by how deeply the biological effect penetrates into the tissue and by how long it endures. It is therefore a less reliable indicator of the presence of a binding-site barrier. A targeted agent that penetrates very poorly into the tissue and delivers a large amount of cargo only to the first cell layers, where the effect is long-lasting, has a similar "inhibitory effect" to that of a targeted agent that delivers cargo more homogenously, whose effect is more spread out and therefore short-lived.

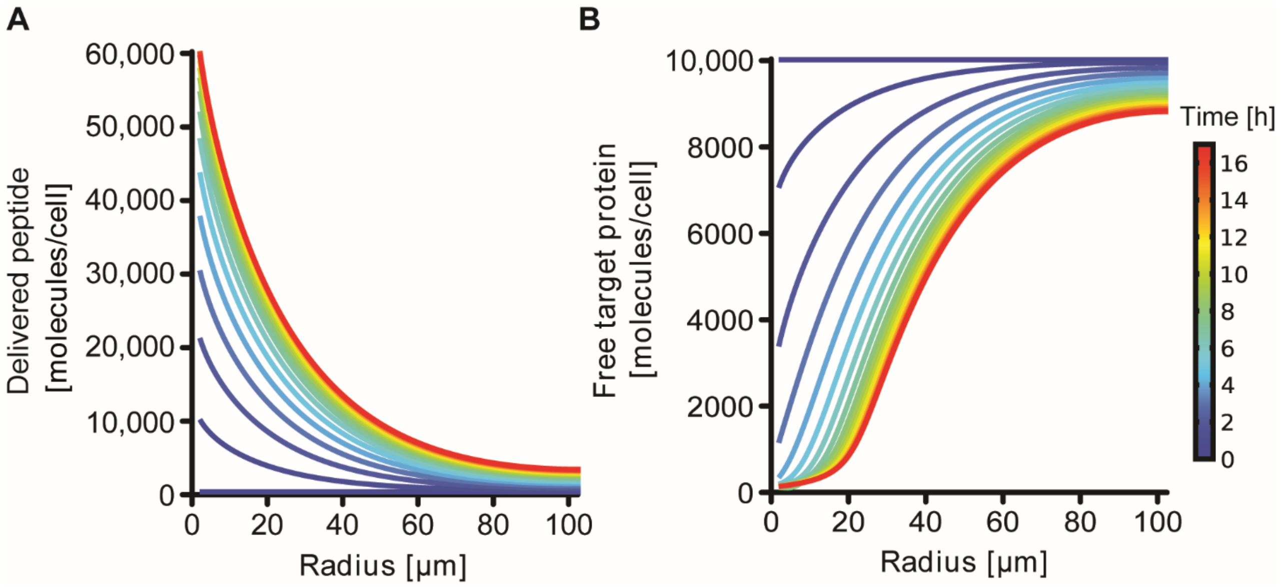

Figure S1. Radius of simulated delivery and biological effect of a targeted peptide. (**A**) Simulation of radial and time-dependency of delivery of a model peptide (3.3 kDa, target affinity: 68 pM) in tumor tissue without convection. Lines represent different timepoints shown up to the point of maximum delivery, 17 h in this instance. (**B**) Depiction of the number of free cytosolic target proteins for the situation where an inhibitory target-binding peptide is delivered under the same conditions as in (**A**). Model assumptions are described in the Supplementary Methods.

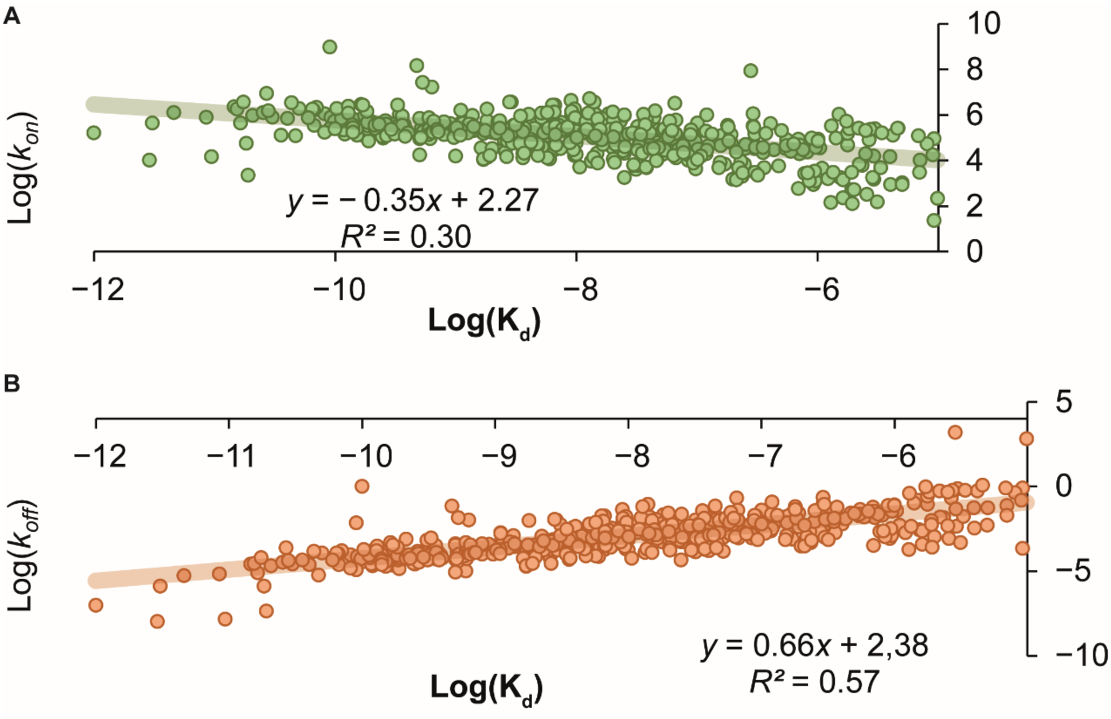

Figure S2. Derivation of k_{on} and k_{off} rates utilized for varying the affinity in simulations. Kinetic data for protein–protein, protein–peptide, and peptide–peptide interactions were selected for affinities in the range of $1.0 \times 10^{12} < K_d$ (M) $< 1.0 \times 10^{-5}$ from the Koffi kinetics database by Norval et al. [26]. Linear regression was used to produce formulas giving the dependency of the decadic logarithm of k_{on} (**A**), and k_{off} (**B**) on the decadic logarithm of K_d.

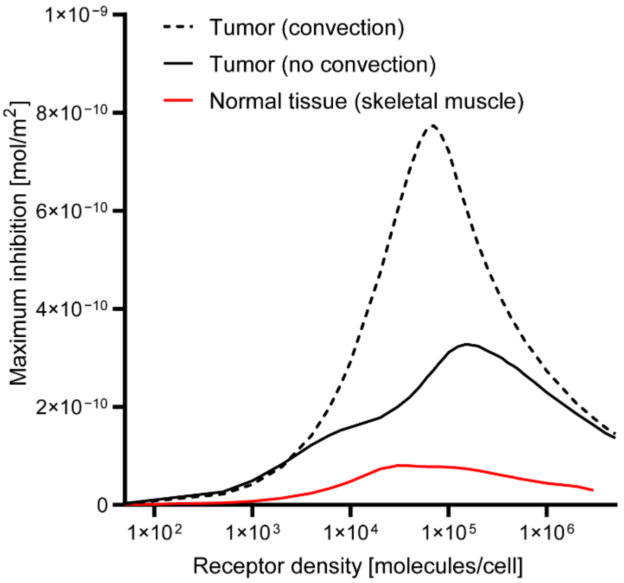

Figure S3. Effect of receptor density on target protein inhibition. For other parameters, default conditions were utilized, as described in Figure 2A. Model assumptions are described in the Supplementary Methods.

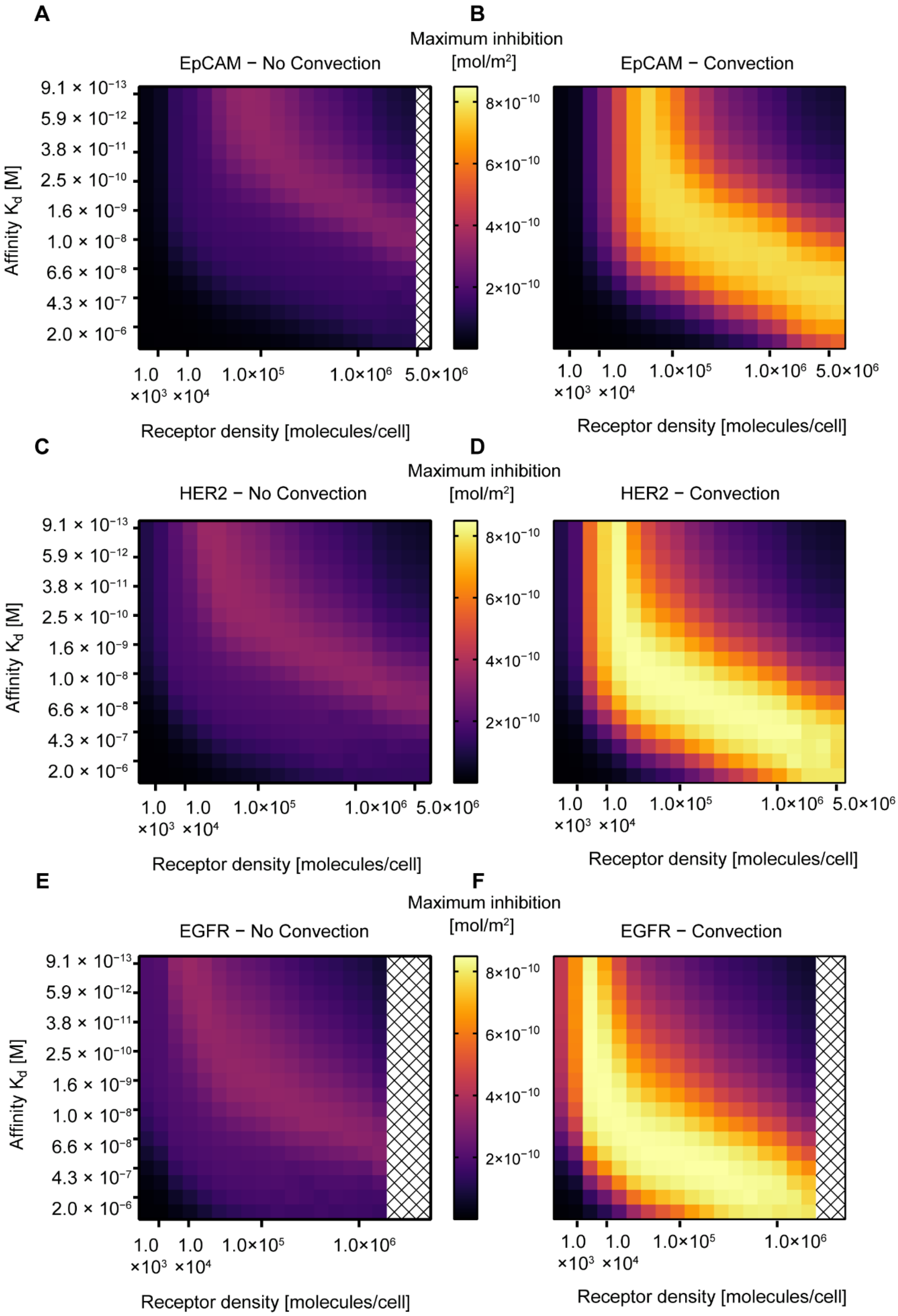

Figure S4. Heatmaps showing the maximum inhibition achieved by targeting receptors. Maximum inhibition was calculated for different expression levels and with targeted agents of varying affinities in tumors with and without convection. (**A**, **B**) Heatmap for a receptor with the internalization rate of EpCAM of 0.002 min^{-1} [18] in tumors without and with convection, respectively; (**C**, **D**) same as (**A, B**), but for a receptor with the internalization rate of HER2 of 0.01 min^{-1} [30]; (**E**,**F**) same as (**A, B**), but for a receptor with the internalization rate of EGFR of 0.08 min^{-1} [30].

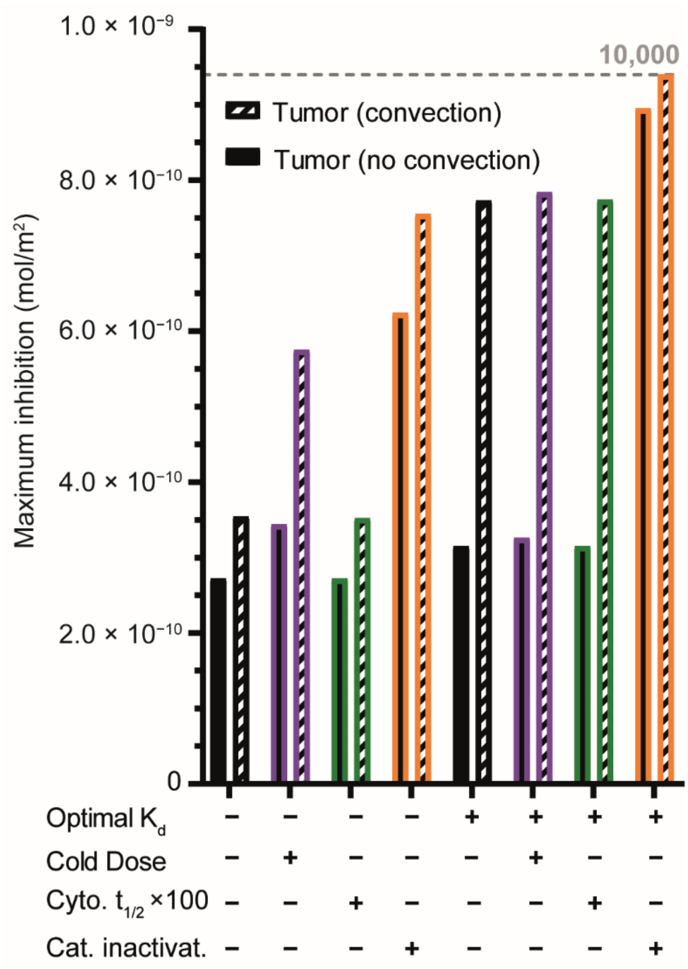

Figure S5. Comparison of the maximum inhibition produced by different strategies. The maximum inhibition of a single dose yielding a plasma concentration of 1 μM was evaluated in tumors that exhibit convection and those that do not. Cyto $t_{1/2}$ × 100 indicates a 100× longer half-life in the cytosol of the targeted agent. Cat. inactivat. refers to catalytic inactivators. Note that the *y*-axis reflects maximum inhibition, which is distinct from the inhibitory effect depicted on the *y*-axis in Figure 5G, as explained in the text.

Table S1. Time-dependent partial differential equations of the model.

TA in tissue	$\underbrace{\frac{\partial TA}{\partial t}}_{\text{TA rate of change}} = \underbrace{D\frac{1}{r}\frac{\partial}{\partial r}\left(r\frac{\partial TA}{\partial r}\right)}_{\text{Diffusion}} - \underbrace{u_{int}\frac{\partial TA}{\partial r}}_{\text{Convection}} - \underbrace{\frac{k_{on}}{\varepsilon}TARe}_{\text{Binding}} + \underbrace{k_{off}B}_{\text{Release}}$	(1)
Free receptor	$\underbrace{\frac{\partial Re}{\partial t}}_{\text{Receptor rate of change}} = \underbrace{k_e Re_0}_{\text{Receptor synthesis}} + \underbrace{k_{off}B}_{\text{Release}} - \underbrace{\frac{k_{on}}{\varepsilon}TARe}_{\text{Binding}} - \underbrace{k_e Re}_{\text{Internalization}}$	(2)
TA–receptor complex	$\underbrace{\frac{\partial B}{\partial t}}_{\text{Bound complex rate of change}} = \underbrace{\frac{k_{on}}{\varepsilon}TARe}_{\text{Binding}} - \underbrace{k_{off}B}_{\text{Release}} - \underbrace{k_e B}_{\text{Internalization}}$	(3)
TA in cytosol	$\underbrace{\frac{\partial TA_{cyto}}{\partial t}}_{\text{TAcyto rate of change}} = \underbrace{Cde\, k_e B \frac{1}{1-\frac{V_{int.}}{V_{total}}}}_{\text{cytosolic import}} + \underbrace{k_{off\,2}I}_{\text{Release}} - \underbrace{k_{on\,2}TA_{cyto}T}_{\text{Binding}} - \underbrace{k_3 TA_{cyto}}_{\text{Degradation}}$	(4)
Target protein	$\underbrace{\frac{\partial T}{\partial t}}_{\text{Target rate of change}} = \underbrace{k_4 T_0}_{\text{Target synthesis}} + \underbrace{k_{off\,2}I}_{\text{Release}} - \underbrace{k_{on\,2}TA_{cyto}T}_{\text{Binding}} - \underbrace{k_4 T}_{\text{Degradation}}$	(5)

TA–target complex in cytosol	$\underbrace{\frac{\partial I}{\partial t}}_{\text{Complex rate of change}} = \underbrace{k_{on\,2} TA_{cyto} T}_{\text{Binding}} - \underbrace{k_{off2} I}_{\text{Release}} - \underbrace{k_5 I}_{\text{Degradation}}$	(6)

The partial differential equations used for the model are given in Table S1. These six equations describe the changes in the free therapeutic protein in the interstitium (1), the unbound cell surface receptor (2), the surface complex of receptor and therapeutic protein (3), the free therapeutic protein delivered to the cytosol (4), the free cytosolic target protein (4), and lastly the inhibitory complex of therapeutic protein and cytosolic target (5).

Table S2. Default parameters (tumor tissue / 70 kDa protein).

Parameter (units)	Value	Reference
$TA_{0\text{-plasma}}$ (nM)	1000 *	[6]
$t_{1/2}(TA_{plasma})$ (h)	2.55	[7]
P (cm/s)	9.8×10^{-7}	[10]
D (cm²/s)	1.9×10^{-7}	[11]
v_{int} (μm/s)	0.55	[12]
ε (n.a.)	0.24	[13,15]
Re_0 (molecules/cell)	5.3×10^{5}	[16]
K_d (M) **	6.8×10^{-11}	[31]
k_{on} ($M^{-1}/^{1}$)**	3.6×10^{5}	[31]
k_{off} (s^{-1}) **	2.4×10^{-5}	[31]
k_e (s^{-1})	3.5×10^{-5}	[18]
$t_{1/2}$ (TA_{cyto}, I, T) (h)	35.5	[19]
V_{cell} (pL)	1.76	[20]
V_{int}/V_{total}	0.5	[21]
Cytosolic delivery efficiency	10% ***	[32]
T_0 (molecules/cell)	10,000 ****	[22]
K_{d2} (M)	1.04×10^{-9}	[17]
k_{on2} (M^{-1}/s^{1})	1.90×10^{5}	[17]
k_{off2} (s^{-1})	1.97×10^{-4}	[17]
k_{cat} (min^{-1})	0.93	[28]

* this value is representative of realistic plasma concentrations reached with monoclonal antibodies;
** values derived from the affinity of Ec1 for epithelial cell adhesion molecules;
*** this value is representative of a highly effective transport system;
**** this value is close to the median target protein concentration in the cytosol.

Table S3. Specific parameters for tissue and molecular weight dependence.

Tissue	MW (kDa) (for permeability estimation)	Microvascular Permeability P (cm/s)	Reference	Interstitial Diffusivity D (cm²/s)	Reference	Plasma Half-life $t_{\frac{1}{2}}$ (h)	Reference	ε
Tumor	3.3	1.5×10^{-5}	[10]	2.5×10^{-6}	[33]	0.28	[34]	0.24
V_{int}/V_{tot} = 0.5 [21]	70	9.8×10^{-7}	[10]	1.9×10^{-7}	[11]	2.55	[7]	0.24
Skeletal muscle	5.5 (Inulin)	1.2×10^{-6}	[36]	4.4×10^{-7}	[37]	0.28	[34]	0.12
V_{int}/V_{tot} = 0.16 [35]	70	1.8×10^{-7}	[38]	0.59×10^{-7}	[37]	2.55	[7]	0.12
Cold dose	10	3.2×10^{-6}	[10]	6.91×10^{-7}	[11]	2.55	[7]	n/a

Table S4. Modified partial differential equations used to model catalytic degradation.

TA in cytosol — catalytic	$\underbrace{\frac{\partial TA_{cyto}}{\partial t}}_{TA\text{-cyto rate of change}} = \underbrace{Cde\; k_e B \frac{1}{1-\frac{V_{int.}}{V_{total}}}}_{\text{Cytosolic import}} + \underbrace{k_{off\,2} I}_{\text{Release}} + \underbrace{k_{cat} I}_{\text{Catalytic degradation}} - \underbrace{k_{on\,2} TA_{cyto} T}_{\text{Binding}} - \underbrace{k_3 TA_{cyto}}_{\text{Degradation}}$	(15)
TA–target complex in cytosol — catalytic	$\underbrace{\frac{\partial I}{\partial t}}_{\text{Complex rate of change}} = \underbrace{k_{on\,2} TA_{cyto} T}_{\text{Binding}} - \underbrace{k_{off2} I}_{\text{Release}} - \underbrace{k_{cat} I}_{\text{Catalytic degradation}} - \underbrace{k_5 I}_{\text{Degradation}}$	(16)

Table S4 gives the modified partial differential equations that were used in order to model an enzymatic cargo. The change in concentration of the free enzymatic therapeutic agent in the cytosol is given by equation 15, and the change in concentration of the complex of therapeutic agent and its substrate, the cytosolic target, is given by equation 16.

Table S5. Partial differential equations used to model cold dosing.

Free cold dose	$\underbrace{\frac{\partial C}{\partial t}}_{\text{Cold dose rate of change}} = \underbrace{D_2 \frac{1}{r}\frac{\partial}{\partial r}\left(r\frac{\partial C}{\partial r}\right)}_{\text{Diffusion}} - \underbrace{u_{int}\frac{\partial C}{\partial r}}_{\text{Convection}} - \underbrace{\frac{k_{con}}{\varepsilon}CRe}_{\text{Binding}} + \underbrace{k_{coff}E}_{\text{Release}}$	(17)
Free receptor	$\underbrace{\frac{\partial Re}{\partial t}}_{\text{Re rate of change}} = \underbrace{k_e Re_0}_{\text{Receptor synthesis}} + \underbrace{k_{off}B}_{\text{Release}} - \underbrace{\frac{k_{on}}{\varepsilon}TARe}_{\text{Binding target}} - \underbrace{k_e Re}_{\text{Internalization}} + \underbrace{k_e E}_{\text{Recycling}} + \underbrace{k_{coff}E}_{\text{Release}} - \underbrace{\frac{k_{con}}{\varepsilon}CRe}_{\text{Binding cold dose}}$	(18)
Cold dose–receptor complex	$\underbrace{\frac{\partial E}{\partial t}}_{\text{Complex rate of change}} = \underbrace{\frac{k_{con}}{\varepsilon}CRe}_{\text{Binding}} - \underbrace{k_{coff}E}_{\text{Release}} - \underbrace{k_e E}_{\text{Internalization}}$	(19)

The additional and modified partial differential required to model cold dosing are given in Table S5. The change in concentration of the cold dose in the interstitium is given by equation 17, the modified partial differential equation giving the change in the targeted receptor is given by equation 18, and finally the changes in the complex formed by the binding of the cold dose to the receptor are given by equation 19.

References

1. Thurber, G.M.; Zajic, S.C.; Wittrup, K.D. Theoretic criteria for antibody penetration into solid tumors and micrometastases. *J. Nucl. Med.* **2007**, *48*, 995–999, doi:10.2967/jnumed.106.037069.
2. Almaca, J.; Weitz, J.; Rodriguez-Diaz, R.; Pereira, E.; Caicedo, A. The Pericyte of the Pancreatic Islet Regulates Capillary Diameter and Local Blood Flow. *Cell. Metab.* **2018**, *27*, 630–644 e634, doi:10.1016/j.cmet.2018.02.016.
3. Krogh, A. The supply of oxygen to the tissues and the regulation of the capillary circulation. *J. Physiol.* **1919**, *52*, 457–474, doi:10.1113/jphysiol.1919.sp001844.
4. Semenza, G.L. Hypoxia, clonal selection, and the role of HIF-1 in tumor progression. *Crit. Rev. Biochem. Mol. Biol.* **2000**, *35*, 71–103, doi:10.1080/10409230091169186.
5. Thurber, G.M.; Wittrup, K.D. Quantitative spatiotemporal analysis of antibody fragment diffusion and endocytic consumption in tumor spheroids. *Cancer Res.* **2008**, *68*, 3334–3341, doi:10.1158/0008-5472.CAN-07-3018.
6. Leveque, D.; Wisniewski, S.; Jehl, F. Pharmacokinetics of therapeutic monoclonal antibodies used in oncology. *Anticancer Res.* **2005**, *25*, 2327–2343.
7. Tang, L.; Persky, A.M.; Hochhaus, G.; Meibohm, B. Pharmacokinetic aspects of biotechnology products. *J. Pharm. Sci.* **2004**, *93*, 2184–2204, doi:10.1002/jps.20125.
8. Usmani, S.S.; Bedi, G.; Samuel, J.S.; Singh, S.; Kalra, S.; Kumar, P.; Ahuja, A.A.; Sharma, M.; Gautam, A.; Raghava, G.P.S. THPdb: Database of FDA-approved peptide and protein therapeutics. *PLoS One* **2017**, *12*, e0181748, doi:10.1371/journal.pone.0181748.
9. Olafsen, T.; Kenanova, V.E.; Sundaresan, G.; Anderson, A.L.; Crow, D.; Yazaki, P.J.; Li, L.; Press, M.F.; Gambhir, S.S.; Williams, L.E.; et al. Optimizing radiolabeled engineered anti-p185HER2 antibody fragments for in vivo imaging. *Cancer Res.* **2005**, *65*, 5907–5916, doi:10.1158/0008-5472.CAN-04-4472.
10. Dreher, M.R.; Liu, W.; Michelich, C.R.; Dewhirst, M.W.; Yuan, F.; Chilkoti, A. Tumor vascular permeability, accumulation, and penetration of macromolecular drug carriers. *J. Natl. Cancer Inst.* **2006**, *98*, 335–344, doi:10.1093/jnci/djj070.
11. Nugent, L.J.; Jain, R.K. Extravascular Diffusion in Normal and Neoplastic Tissues. *Cancer Res.* **1984**, *44*, 238–244.
12. Chary, S.R.; Jain, R.K. Direct measurement of interstitial convection and diffusion of albumin in normal and neoplastic tissues by fluorescence photobleaching. *Proc Natl. Acad. Sci. U S A* **1989**, *86*, 5385–5389, doi:10.1073/pnas.86.14.5385.
13. Wiig, H.; Gyenge, C.C.; Tenstad, O. The interstitial distribution of macromolecules in rat tumours is influenced by the negatively charged matrix components. *J. Physiol.* **2005**, *567*, 557-567, doi:10.1113/jphysiol.2005.089615.
14. Schmidt, M.M.; Wittrup, K.D. A modeling analysis of the effects of molecular size and binding affinity on tumor targeting. *Mol. Cancer Ther.* **2009**, *8*, 2861–2871, doi:10.1158/1535-7163.MCT-09-0195.
15. Vasalou, C.; Helmlinger, G.; Gomes, B. A mechanistic tumor penetration model to guide antibody drug conjugate design. *PLoS One* **2015**, *10*, e0118977, doi:10.1371/journal.pone.0118977.
16. Chernyavska, M.; Schmid, M.; Freitag, P.C.; Palacio-Castañeda, V.; Piruska, A.; Huck, W.T.S.; Plückthun, A.; Verdurmen, W.P.R. Unravelling Receptor and RGD Motif Dependence of Retargeted Adenoviral Vectors using Advanced Tumor Model Systems. *Sci. Rep.* **2019**, *9*, 18568, doi:10.1038/s41598-019-54939-9.
17. Schilling, J.; Schöppe, J.; Plückthun, A. From DARPins to LoopDARPins: novel LoopDARPin design allows the selection of low picomolar binders in a single round of ribosome display. *J Mol. Biol.* **2014**, *426*, 691–721, doi:10.1016/j.jmb.2013.10.026.
18. Hazin, J.; Moldenhauer, G.; Altevogt, P.; Brady, N.R. A novel method for measuring cellular antibody uptake using imaging flow cytometry reveals distinct uptake rates for two different monoclonal antibodies targeting L1. *J. Immunol. Methods.* **2015**, *423*, 70–77, doi:10.1016/j.jim.2015.04.024.

19. Cambridge, S.B.; Gnad, F.; Nguyen, C.; Bermejo, J.L.; Kruger, M.; Mann, M. Systems-wide proteomic analysis in mammalian cells reveals conserved, functional protein turnover. *J. Proteome Res.* **2011**, *10*, 5275–5284, doi:10.1021/pr101183k.
20. Gamcsik, M.P.; Millis, K.K.; Colvin, O.M. Noninvasive detection of elevated glutathione levels in MCF-7 cells resistant to 4-hydroperoxycyclophosphamide. *Cancer Res.* **1995**, *55*, 2012–2016.
21. Krol, A.; Maresca, J.; Dewhirst, M.W.; Yuan, F. Available volume fraction of macromolecules in the extravascular space of a fibrosarcoma: implications for drug delivery. *Cancer Res.* **1999**, *59*, 4136–4141.
22. de Godoy, L.M.; Olsen, J.V.; Cox, J.; Nielsen, M.L.; Hubner, N.C.; Frohlich, F.; Walther, T.C.; Mann, M. Comprehensive mass-spectrometry-based proteome quantification of haploid versus diploid yeast. *Nature* **2008**, *455*, 1251–1254, doi:10.1038/nature07341.
23. Holtz, J. *Comprehensive Human Physiology.* Springer, 1; Springerg: Berlin, Germany, **1996**; 1917–1939.
24. Owen-Woods, C.; Joulia, R.; Barkaway, A.; Rolas, L.; Ma, B.; Nottebaum, A.F.; Arkill, K.P.; Stein, M.; Girbl, T.; Golding, M.; et al. Local microvascular leakage promotes trafficking of activated neutrophils to remote organs. *J. Clin. Invest.* **2020**, *130*, 2301–2318, doi:10.1172/JCI133661.
25. Minchinton, A.I.; Tannock, I.F. Drug penetration in solid tumours. *Nat. Rev. Cancer* **2006**, *6*, 583–592, doi:10.1038/nrc1893.
26. Norval, L.W.; Kramer, S.D.; Gao, M.; Herz, T.; Li, J.; Rath, C.; Wohrle, J.; Gunther, S.; Roth, G. KOFFI and Anabel 2.0-a new binding kinetics database and its integration in an open-source binding analysis software. *Database (Oxford)* **2019**, *2019*, doi:10.1093/database/baz101.
27. Chopra, R.; Sadok, A.; Collins, I. A critical evaluation of the approaches to targeted protein degradation for drug discovery. *Drug Discov. Today Technol.* **2019**, *31*, 5–13, doi:10.1016/j.ddtec.2019.02.002.
28. Saha, A.; Deshaies, R.J. Multimodal activation of the ubiquitin ligase SCF by Nedd8 conjugation. *Mol. Cell* **2008**, *32*, 21–31, doi:10.1016/j.molcel.2008.08.021.
29. Scott, D.C.; Rhee, D.Y.; Duda, D.M.; Kelsall, I.R.; Olszewski, J.L.; Paulo, J.A.; de Jong, A.; Ovaa, H.; Alpi, A.F.; Harper, J.W.; et al. Two Distinct Types of E3 Ligases Work in Unison to Regulate Substrate Ubiquitylation. *Cell* **2016**, *166*, 1198–1214 e1124, doi:10.1016/j.cell.2016.07.027.
30. Hendriks, B.S.; Opresko, L.K.; Wiley, H.S.; Lauffenburger, D. Quantitative analysis of HER2-mediated effects on HER2 and epidermal growth factor receptor endocytosis: distribution of homo- and heterodimers depends on relative HER2 levels. *J. Biol. Chem.* **2003**, *278*, 23343-23351, doi:10.1074/jbc.M300477200.
31. Stefan, N.; Martin-Killias, P.; Wyss-Stoeckle, S.; Honegger, A.; Zangemeister-Wittke, U.; Plückthun, A. DARPins recognizing the tumor-associated antigen EpCAM selected by phage and ribosome display and engineered for multivalency. *J. Mol. Biol.* **2011**, *413*, 826–843, doi:10.1016/j.jmb.2011.09.016.
32. Verdurmen, W.P.R.; Mazlami, M.; Plückthun, A. A quantitative comparison of cytosolic delivery via different protein uptake systems. *Sci. Rep.* **2017**, *7*, 13194, doi:10.1038/s41598-017-13469-y.
33. Chakraborty, S.; Ozkan, A.; Rylander, M.N.; Woodward, W.A.; Vlachos, P. Mixture theory modeling for characterizing solute transport in breast tumor tissues. *J. Biol. Eng.* **2019**, *13*, 46, doi:10.1186/s13036-019-0178-z.
34. Buclin, T.; Cosma Rochat, M.; Burckhardt, P.; Azria, M.; Attinger, M. Bioavailability and biological efficacy of a new oral formulation of salmon calcitonin in healthy volunteers. *J. Bone. Miner .Res.* **2002**, *17*, 1478–1485, doi:10.1359/jbmr.2002.17.8.1478.
35. Kim, Y.R.; Savellano, M.D.; Savellano, D.H.; Weissleder, R.; Bogdanov, A., Jr. Measurement of tumor interstitial volume fraction: method and implication for drug delivery. *Magn. Reson. Med.* **2004**, *52*, 485–494, doi:10.1002/mrm.20182.
36. Simionescu, N.; Simionescu, M. *Endothelial cell biology in health and disease*; Plenum Press: New York, USA, 1988; pp. xvii, 458 p.
37. Shi, L.; Zeng, M.; Sun, Y.; Fu, B.M. Quantification of blood-brain barrier solute permeability and brain transport by multiphoton microscopy. *J. Biomech. Eng.* **2014**, *136*, doi:10.1115/1.4025892.
38. Kim, D.; Armenante, P.M.; Duran, W.N. Transient analysis of macromolecular transport across microvascular wall and into interstitium. *Am. J. Physiol.* **1993**, *265*, H993–999, doi:10.1152/ajpheart.1993.265.3.H993.
